# Normal sulfation levels regulate spinal cord neural precursor cell proliferation and differentiation

**DOI:** 10.1186/1749-8104-7-20

**Published:** 2012-06-08

**Authors:** Michael Karus, Samira Samtleben, Claudia Busse, Teresa Tsai, Irmgard D Dietzel, Andreas Faissner, Stefan Wiese

**Affiliations:** 1 Department of Cell Morphology and Molecular Neurobiology, Ruhr-University Bochum, Bochum, Germany; 2 Group for Molecular Cell Biology, Ruhr-University Bochum, Bochum, Germany; 3 Department of Neurobiochemistry, Ruhr-University Bochum, Bochum, Germany; 4 International Graduate School of Neuroscience, Ruhr-University Bochum, Bochum, Germany

**Keywords:** Chondroitin sulfate proteoglycans, Extracellular matrix, Neuronal differentiation, Sulfation

## Abstract

**Background:**

Sulfated glycosaminoglycan chains are known for their regulatory functions during neural development and regeneration. However, it is still unknown whether the sulfate residues alone influence, for example, neural precursor cell behavior or whether they act in concert with the sugar backbone. Here, we provide evidence that the unique 473HD-epitope, a representative chondroitin sulfate, is expressed by spinal cord neural precursor cells *in vivo* and *in vitro*, suggesting a potential function of sulfated glycosaminoglycans for spinal cord development.

**Results:**

Thus, we applied the widely used sulfation inhibitor sodium chlorate to analyze the importance of normal sulfation levels for spinal cord neural precursor cell biology *in vitro*. Addition of sodium chlorate to spinal cord neural precursor cell cultures affected cell cycle progression accompanied by changed extracellular signal-regulated kinase 1 or 2 activation levels. This resulted in a higher percentage of neurons already under proliferative conditions. In contrast, the relative number of glial cells was largely unaffected. Strikingly, both morphological and electrophysiological characterization of neural precursor cell-derived neurons demonstrated an attenuated neuronal maturation in the presence of sodium chlorate, including a disturbed neuronal polarization.

**Conclusions:**

In summary, our data suggest that sulfation is an important regulator of both neural precursor cell proliferation and maturation of the neural precursor cell progeny in the developing mouse spinal cord.

## Background

Complex carbohydrate moieties attached either to secreted extracellular matrix molecules or to membrane bound cell adhesion molecules are important players for central nervous system (CNS) development, because they can bind a multitude of cytokines or growth factors [1-3]. Among these carbohydrate structures, the chondroitin sulfate (CS) glycosaminoglycans (GAGs) have been proved to be particularly important for neural precursor cell (NPC) biology, because interference with CS-GAG biology using the bacterial enzyme chondroitinase ABC (ChABC) strongly affects NPC proliferation and differentiation [4-7]. CS-GAGs are highly sulfated, and the sulfation pattern results from the specific expression pattern of various CS-sulfotransferases during CNS development and in adulthood [8]. Sulfated CS-GAGs also play a role in pathophysiological situations. Up-regulation of CS proteoglycans (PGs) bearing complex CS-GAGs after CNS injury is associated with a specific sulfation pattern on CS-GAGs, mediating potentially inhibitory properties of PGs on axonal regeneration [9,10]. These inhibitory properties can be overcome using ChABC in combination with defined motor tasks [11,12]. However, different sulfation patterns appear to differentially affect axonal regeneration after CNS lesion [13].

Besides CS-GAGs, the heparan sulfate (HS)-GAGs constitute a second major class of sulfated glycans in the CNS. HS-GAGs are known for their strong impact on fibroblast growth factor (FGF) and Sonic hedgehog signaling [14]. In addition, some other glycan structures can be sulfated as well. Among these, the complex LewisX glycan, which is expressed by NPCs during development and in adulthood [15-17], and the human natural killer 1 antigen are the most prominent ones [18].

The transfer of sulfate on the glycosaminoglycan backbone of CS-GAGs and HS-GAGs is catalyzed by CS- or HS-specific sulfotransferases located in the Golgi apparatus [19,20]. 3′-phosphoadenosine 5′-phosphosulfate (PAPS) serves as the sulfate donor. The enzymatic generation of PAPS can be inhibited using sodium chlorate (NaClO_3_) [21], resulting in hypo-sulfated GAG chains. Therefore, NaClO_3_ is often used to pharmacologically interfere with GAG biology. Here, we used NaClO_3_ to further elucidate the role of sulfation for differentiation and proliferation of spinal cord NPCs cultivated as free floating neurospheres. Neurospheres treated with NaClO_3_ showed a reduction in growth accompanied by changes in neuronal differentiation and maturation. In contrast, differentiation towards glial lineages seemed to be largely unaffected. Thus, we propose that the proliferation of spinal cord NPCs and the maturation of spinal cord NPC-derived neurons depend on a specific sulfation pattern during development.

## Methods

### Animals

All experiments were performed according to international rules using timed-pregnant wild-type NRMI mice kept under standard housing conditions. The age of the embryos was determined following the Theiler Stages, and the day of the vaginal plug was considered as E0.5.

### Neurosphere culture

Cultivation of mouse spinal cord NPCs was carried out as previously described [22]. Briefly, the spinal cords of E13.5 to E18.5 mouse embryos were dissected using small forceps. Afterwards, the isolated spinal cords were enzymatically dissociated with 30 U/mL Papain (Worthington, New Jersey, USA), resulting in a single cell suspension. After centrifugation for 5 min at 200 g the cell sediment was resuspended in neurosphere medium containing DMEM and nutrient mixture F12 (in a ratio of 1:1; both Gibco, Karlsruhe, Germany), 2 % (v/v) B27 (Invitrogen, Karlsruhe, Germany), 1 % (v/v) L-glutamine (Invitrogen) and 1 % (v/v) penicillin/streptomycin (Invitrogen). Under proliferative conditions, either 1,250 cells/mL (clonal density) or 100,000 cells/mL (high density culture) were plated, and either 10 ng/mL (clonal density) or 20 ng/mL (high density culture) EGF and FGF2 (both PreproTech, Rocky Hill, USA) were added. FGF2 containing cultures were additionally supplemented with 0.25 U/mL (clonal density) or 0.5 U/mL (high density culture) heparin (Sigma-Aldrich, Munich, Germany). After one week at 37°C and 5 % (v/v) CO_2_, neurospheres had formed. For clonal analyses, the cells were kept for one week without any agitation, in order to avoid neurosphere fusion events. As recently described, 30 mM NaClO_3_ or the respective solvent were added for the entire cultivation period [23].

For differentiation analyses, primary neurospheres were dissociated using trypsin/ethylenediaminetetraacetic acid (EDTA; Invitrogen) for 4 min at 37°C, and single cells were plated at 5,000 cells/well onto poly-DL-ornithine/laminin coated four-well dishes (Greiner, Frickenhausen, Germany) for another 4 days in the presence of 1 % (v/v) FCS and either 30 mM NaClO_3_ or the respective solvent. Afterwards, the cells were either subjected to immunocytochemical or electrophysiological analyses.

### Immunological reagents

In the following the primary antibodies and the respective dilutions used in this study are listed. The monoclonal antibodies were: anti-O4 (1:50; mouse IgM) [24], anti-Nestin (1:500; mouse IgG; clone rat-401; Chemicon, Hofheim, Germany), anti-α-Tubulin (1:10,000; mouse IgG; clone DM1a; Sigma-Aldrich), anti-βIII-Tubulin (1:500; mouse IgG; clone SDL3D10; Sigma-Aldrich) and anti-DSD-1-epitope (1:300 (immunofluorescence), 1:100 (western blot); rat IgM; clone 473) [25]. The polyclonal antibodies were: anti-GFAP (1:300; rabbit; Dako, Hamburg, Germany), anti-EGFR (1:500; rabbit; Santa Cruz, Hamburg, Germany), anti- receptor protein tyrosine phosphatase (RPTP)-β/ζ (1:300 (immunofluorescence), 1:1000 (western blot); rabbit; batch Kaf13/5) [25], anti-BLBP (1:300; rabbit; Chemicon), anti-GLAST (1:1000; guinea-pig; Chemicon), anti-pH3 (1:300; rabbit; Chemicon), anti-Erk1/2 (1:1000; rabbit; Santa Cruz), anti-pErk1/2 (1:1000; rabbit; Cell Signaling Technologies, Beverly, USA), anti-Akt (1:1000; rabbit; Cell Signaling Technology), anti-pAkt (1:1000; rabbit; Cell Signaling Technologies), anti-MAP2 (1:300: rabbit; Chemicon) and anti-Tau (1:300; mouse IgG) [26].

### Immunohistochemistry

Pregnant mice were killed by cervical dislocation, and the embryos were immediately removed. The embryos’ trunks were washed once with PBS and then fixed in 4 % (w/v) paraformaldehyde (PFA) at 4°C. Depending on the age of the embryo, the fixation time varied between 30 min (E9.5) and 20 h (E18.5). After fixation the embryos were transferred to 20 % (w/v) sucrose for cryoprotection. Finally, the tissue was embedded in Tissue Tec Freezing Medium (Jung, Nussloch, Germany) and cut into 16 μm thin sections on a cryostat CM3050S (Leica, Solms, Germany). The sections were mounted on superfrost slides (Thermo Scientific, Schwerte, Germany) and stored at −20°C until further use.

For immunohistochemical analyses of neurospheres, the neurospheres were transferred into a 1.5 mL Eppendorf tube, briefly washed with PBS and then fixed for 1 h in 4 % (w/v) PFA at 4°C. Subsequently, the PFA was removed, and 20 % (w/v) diethylpyrocarbonate-treated sucrose was added for cryoprotection for 4 h at 4°C. Finally, the neurospheres were embedded using Tissue Tec Freezing Medium and cut into 16 μm thin cryosections on a cryostat CM3050S. The cryosections were rehydrated and blocked for 1 h at room temperature with PBT1 (PBS + 1 % (w/v) BSA + 0.1 % (v/v) Triton X-100) and 1.7 % (w/v) NaCl-PBS (PBS + 0.9 % (w/v) NaCl) in a ratio of 1:1 plus 10 % (v/v) normal goat serum, followed by incubation with the primary antibodies diluted in PBT1 + 5 % (v/v) normal goat serum overnight at 4°C. The next day, the sections were washed three times with PBS and subsequently incubated with species-specific antibodies coupled with either Cy2 (1:250) or Cy3 (1:500) (Dianova, Hamburg, Germany) diluted in PBS/A (PBS + 0.1 % (w/v) BSA) for 3 h at room temperature. Hoechst 33528 (Sigma) was included (diluted 1:10^5^ in PBS), to additionally label the nuclei. The sections were washed three times with PBS and finally mounted with ImmuMount (Invitrogen).

### Immunocytochemistry

After removal of the culture medium, adherent cells were briefly washed twice with PBS/A. In case of membrane bound or extracellular epitopes (O4, 473HD), the incubation with the primary antibody diluted in PBS/A was directly carried out for 30 min at room temperature. Then, the cells were washed again three times with PBS/A and fixed with 4 % (w/v) PFA for 10 min at room temperature. To detect intracellular epitopes, the fixation was performed prior to the incubation with the primary antibodies diluted in PBT1. After incubation with the primary antibody, the cells were washed three times with PBT1 and the incubation with either Cy3- or Cy2- or HRP (1:500)-coupled species-specific secondary antibodies (Dianova) diluted in PBS/A was carried out at room temperature for 30 min. Hoechst 33528 (1:10^5^) was additionally added to visualize the nuclei. Finally, the cells were washed twice with PBS and mounted in PBS and glycerine (2:1). For BrdU incorporation analysis, 1 μM BrdU was added to the neurospheres for 1 h. Then, the neurospheres were dissociated, and single cells were plated on a poly-DL-ornithine substrate for two hours. Finally, the BrdU-immunocytochemistry was carried out using the BrdU-labeling and detection Kit I (Roche, Mannheim, Germany) according to the manufacturer’s instructions. The diaminobenzidin staining was carried out by incubating the cells with freshly prepared diaminobenzidin (Sigma-Aldrich) diluted in double distilled water for 10 min after incubation with the HRP-coupled secondary antibody. Finally, the cells were washed twice with double distilled water and mounted in PBS and glycerine (2:1).

### Western blot

Neurospheres were homogenized and solubilized by mechanical agitation in 4°C cold cell lysis buffer (50 mM Tris–HCl pH 7.4; 150 mM NaCl; 5 mM EDTA; 5 mM ethyleneglycotetraacetic acid; 1 % (v/v) Triton X100; 0.1 % (v/v) Na-desoxycholate; 0.1 % (v/v) SDS) and incubated for further 30 min on ice. The lysate was then cleared by centrifugation (16,000 g) at 4°C. The protein concentration was determined using a protein quantification kit (Pierce, Rockford, USA) according to the manufacturer’s instructions. 10 μg protein was separated on a 7 % (v/v) SDS-gel and transferred to a PVDF membrane (Roth, Karlsruhe, Germany). After transfer the membrane was blocked with 5 % (w/v) skim milk powder in Tris-buffered-Saline (TBS) for one hour at room temperature. The primary antibodies were diluted in 5 % (w/v) skim milk powder in TBS + 0.05 % (v/v) Tween20 (TBST) and incubated at 4°C over night. Subsequently, the membrane was washed three times with TBST for 10 min and the incubation with the HRP-coupled secondary antibodies (1:5000) diluted in 5 % (w/v) skim milk powder in TBST was carried out at room temperature for one hour. Finally, the membrane was washed again three times with TBS, and the signal was detected using enhanced chemiluminescence reagent (Pierce, Rockfort, USA).

### Electrical recordings

Whole cell patch-clamp recordings of spinal cord NPC-derived neurons were performed using borosilicate glass pipettes of a mean resistance of 2–6 MΩ. The glass pipettes were filled with a solution, mimicking the intracellular ion concentrations (100 mM K-gluconate, 0.1 mM CaCl_2_, 1.1 mM EDTA, 5 mM MgCl_2_, 5 mM NaCl, 10 mM HEPES, 3 mM MgCl_2_-ATP, pH 7.35, 235 mOsm). For the experiments, the cell culture medium was removed and the cells were washed twice with the bath solution (110 mM NaCl, 5.4 mM KCl, 1.8 mM CaCl_2_, 0.8 mM MgCl_2_, 10 mM HEPES, 10 mM glucose, pH 7.35, 250 mOsm) and finally kept in the bath solution at room temperature for no longer than 1 h. The setup was equipped with an inverse microscope including phase contrast optics. The data were acquired using an EPC7 patch-clamp amplifier and processed via Pclamp 6 software. After liquid junction potential correction, the cell was depolarized from −77 mV to 53 mV in 5 mV steps, in order to analyze both the mixed sodium and potassium current density. The cell capacitance was calculated from the integral of the charging curve after application of a step depolarization of 10 mV amplitude. The sodium current was determined from the peak amplitude for a step depolarization to −12 mV. The potassium current was determined after 200 ms of depolarization to 28 mV. To account for the cell size, the calculated currents were normalized to the cell capacitance. Thus, the current densities data are displayed in the units A/F. The data were pooled from five independent preparations. In total, 29 control cells and 31 NaClO_3_ treated cells were analyzed. Each cell with a leak current of more than 20 pA was excluded from the final data analysis, resulting in 12 control cells and 16 NaClO_3_ treated cells.

### Documentation and data analysis

Pictures were taken at an Axioplan2 microscope with the AxioCam HRc camera using the AxioVision 4.4 and 4.5 software (Zeiss, Jena, Germany). For quantitative analyses of immunocytochemical antigen detections, a minimum of 200 Bisbenzimid-positive nuclei were counted in at least three independent experiments per antibody and culture condition.

To quantify the neurosphere formation from primary spinal cord cells, the number of all neurospheres in the whole culture flask was counted after one week. In order to prevent the formation of non-clonal neurospheres, the culture flasks were not taken out of the incubator during the cultivation period. The diameters of single neurospheres as well as the neuronal morphology were analyzed using ImageJ v1.41. Unless otherwise stated, the data are expressed as mean ± SD. Statistical significance was assessed using the paired and unpaired two-tailed Student’s *t* test and the *P*-values are given as *P ≤0.05, **P ≤0.01, and ***P ≤0.001.

## Results

### The sulfation-dependent 473HD-epitope is expressed by neural precursor cells in the embryonic mouse spinal cord

**Figure 1:**
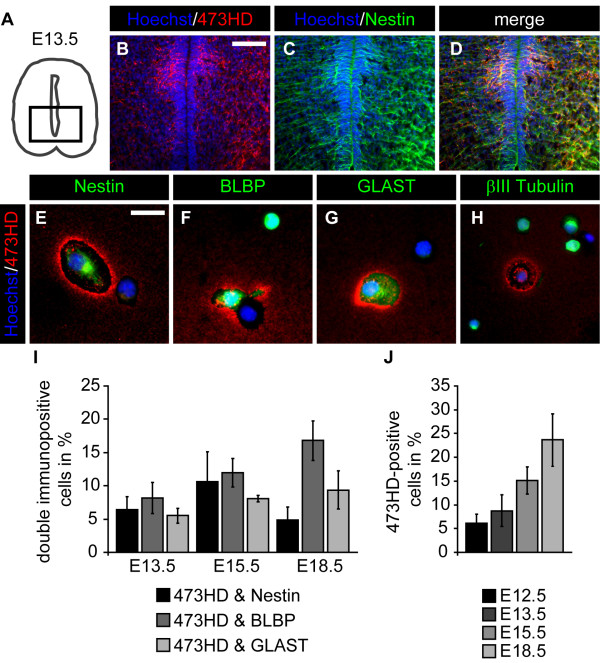
**The 473HD epitope is expressed by neural precursor cells during embryonic mouse spinal cord development. (A)** Schematic drawing of frontal E13.5 spinal cord sections, illustrating the spinal cord region shown in B-D. **(B-D)** Photomicrographs of frontal spinal cord sections stained against the 473HD- epitope and the NPC marker Nestin, showing the colocalization of the 473HD- epitope with Nestin-positive NPC processes around the central canal. **(E-H)** Photomicrographs of acutely dissociated spinal cord cells stained against the 473HD-epitope in combination with either the NPC markers Nestin, BLBP and GLAST or the neuronal marker βIII-Tubulin. The 473HD-epitope was exclusively expressed by NPCs. In contrast, we never observed any neuronal expression of the 473HD-epitope. **(I)** Quantification of double immunopositive cells. At E13.5, the percentage of 473HD-positive cells that also expressed one of the NPC markers was relatively low. The relative amount of 473HD-and-Nestin-positive cells peaked at E15.5. In contrast the 473HD-and-BLBP-positive and the 473HD-and-GLAST-positive populations gradually increased towards the end of embryogenesis. **(J)** Quantification of 473HD-positive cells throughout embryonic spinal cord development, showing the gradual increase of 473HD-positive cells between E12.5 and E18.5. To visualize the nuclei, the cells were counterstained with Hoechst 33528. Scale bar: 50 μm (B-D), 25 μm (E-H).

Since systematic data concerning the expression of GAG chains throughout spinal cord development are missing, we started with an expression analysis of the 473HD-epitope, a representative CS-GAG present on the chondroitin sulfate proteoglycan phosphacan/RPTPβ/ζ [25,27]. Immunohistochemical detection of the 473HD-epitope on frontal spinal cord sections between E9.5 and E18.5 show its up-regulation towards the end of neurogenesis at E12.5 (Additional file 1: Figure S1A, B). The expression was particularly high in the ventral spinal cord between E13.5 and E15.5 (Additional file 1: Figure S1C, D). Towards the end of embryogenesis at E18.5, the 473HD-epitope could be detected within the whole spinal cord except for the central canal region (Additional file 1: Figure S1E). Further immunohistochemical analyses on frontal E13.5 spinal cord sections (Figure 1A) revealed that the 473HD-epitope was expressed by Nestin-positive NPCs *in vivo* (Figure 1B-D). Note that most of the ventricular zone lacks immunoreactivity for the 473HD-epitope except for a distinct region within the ventral spinal cord. To investigate the cellular source, we dissociated the spinal cord from various embryonic ages and plated single cells in low density for two hours on a poly-DL-ornithine substrate. After that, we immunocytochemically characterized the cells using various cell type specific markers. We observed that many 473HD-positive cells co-expressed the NPC markers Nestin, BLBP and GLAST (Figure 1E-G). In contrast, we never observed 473HD immunoreactivity on βIII-Tubulin-positive young neurons (Figure 1H). We further quantified the relative number of 473HD-positive cells expressing the NPC markers Nestin, BLBP and GLAST at E13.5, E15.5 and E18.5. Our findings are summarized in Table 1. The percentage of 473HD-positive cells co-expressing one of the mentioned markers was about 5 % for each marker at E13.5 but increased within the next two days to around 10 % (Figure 1I and Table 1). Towards the end of embryogenesis at E18.5, the percentage of Nestin- and-473HD-positive cells decreased again, while the BLBP-and-473HD populations increased and the GLAST-and-473HD populations did not change (Figure 1I and Table 1). Finally, we determined the overall 473HD-positive cell population throughout development and found a general increase in the relative amount of 473HD-positive cells between E12.5 and E18.5, consistent with our immunohistochemical analyses (E12.5: 6.2 ± 1.9 % (n = 4); E13.5: 9.0 ± 3.3 % (n = 10); E15.5: 15.2 ± 2.7 % (n = 10); E18.5: 23.2 ± 5.3 % (n = 8); Figure 1J).

**Table 1:** Immunocytochemical characterization of 473HD-positive spinal cord cells in the embryonic spinal cord

**Marker**	**E12.5**	**E13.5**	**E15.5**	**E18.5**
**473HD**	6.2 ± 1.9% (n = 4)	9.0 ± 3.3% (n = 10)	15.2 ± 2.7% (n = 10)	23.2 ± 5.3% (n = 8)
**Nestin/473HD**	ND	6.4 ± 2.0% (n = 6)	10.6 ± 4.5% (n = 3)	4.9 ± 1.9% (n = 4)
**BLBP/473HD**	ND	8.7 ± 2.4% (n = 5)	12.7 ± 2.4% (n = 5)	17.3 ± 2.6% (n = 4)
**GLAST/473HD**	ND	6.8 ± 2.8% (n = 4)	9.0 ± 2.1% (n = 5)	9.7 ± 2.4% (n = 4)

The data are depicted as mean ± SD. ND: not determined.

### Sodium chlorate efficiently reduces the level of the sulfation-dependent 473HD-epitope in spinal cord neural precursor cell cultures

**Figure 2:**
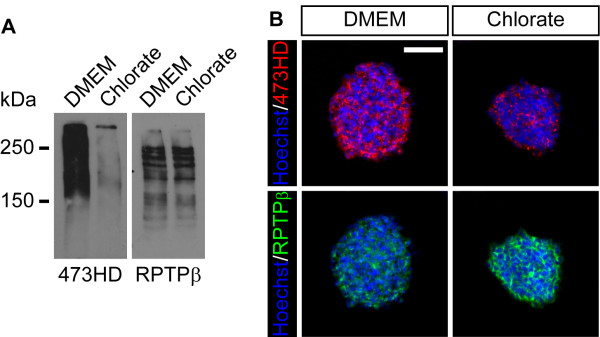
**Sodium chlorate efficiently reduces the expression level of the sulfation-dependent 473HD-epitope. (A)** Western blot analysis of neurosphere detergent extracts, showing a reduced 473HD expression level upon NaClO_3_ treatment. The expression level of different RPTPβ/ζ isoforms, known 473HD carrier proteins, did not appear to be changed in the presence of NaClO_3_. **(B)** Immunohistochemical detection of the 473HD-epitope and RPTPβ/ζ isoforms on neurosphere cryosections confirmed the reduced 473HD expression levels in NaClO_3_-treated cultures. Here, the immunoreactivity for the 473HD carrier proteins seemed to be enhanced. Hoechst 33528 was used to visualize the cell nuclei. Scale bar: 50 μm.

Several studies dealing with GAG biology were based on the usage of NaClO_3_, in order to interfere with the sulfation levels of the GAG chains. In this study we applied NaClO_3_and asked whether alterations in sulfation levels might regulate proliferation, survival and differentiation of spinal cord NPCs grown as free floating neurospheres. We cultured primary neurospheres from E13.5 spinal cord cells and analyzed the expression of the sulfation-dependent 473HD-epitope and its carrier protein RPTPβ/ζ after one week. Western blot analyses of neurosphere detergent extracts revealed that neurospheres expressed high levels of the 473HD-epitope under standard culture conditions. The addition of NaClO_3_ strongly reduced the 473HD levels in comparison to the solvent control (Figure 2A). However, the expression levels of its carrier protein itself appeared not to be affected (Figure 2A). In an independent experiment, neurosphere cryosections were labeled for the 473HD-epitope as well as RPTPβ/ζ. Consistent with the western blot analysis, the 473HD-immunoreactivity was strongly reduced when sulfation levels were decreased using NaClO_3_ (Figure 2B). The RPTPβ/ζ immunoreactivity, however, seemed to be enhanced, potentially reflecting an enhanced accessibility of the epitope rather than an increased expression level (Figure 2B), since the western blot analysis did not reveal changes in the RPTPβ/ζ expression. Yet, these data demonstrate the suitability of NaClO_3_ to suppress normal sulfation levels in spinal cord NPC cultures.

### Sodium chlorate affects spinal cord neural precursor cell growth

**Figure 3:**
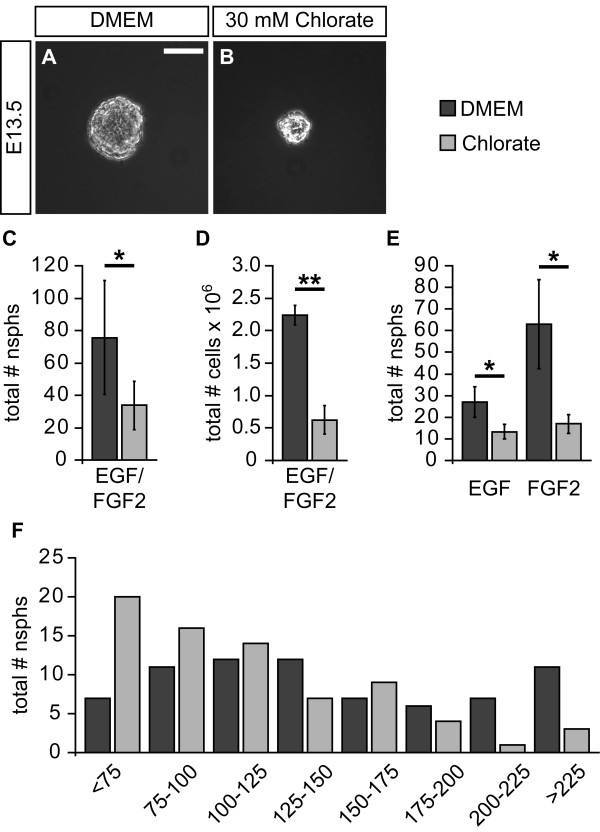
**Sodium chlorate affects clonal neurosphere formation from primary spinal cord cells. (A, B)** Neurospheres were grown under clonal conditions in the presence of EGF and FGF2. The addition of NaClO_3_ resulted in smaller neurospheres in comparison to the solvent control. **(C)** Moreover, the quantification of neurospheres revealed a significantly reduced number of neurospheres in the presence of NaClO_3_ (n = 5; *P* <0.05). **(D)** Consistent with this observation, the total cell number in high density cultures was strongly reduced upon NaClO_3_ treatment after one week (n = 4; *P* <0.01). **(E)** Analysis of EGF- and FGF2-dependent neurosphere formation showed that, under both growth factor conditions, the addition of NaClO_3_ resulted in a significantly smaller number of neurospheres (n = 3; *P* <0.05 for both conditions). **(F)** Using neurosphere size, based on the neurosphere diameter, in the presence or absence of NaClO_3,_ the neurospheres were sorted into different categories. The size distribution histogram shows that NaClO_3_ treatment led to a higher number of small neurospheres. However, some large neurospheres were still detectable. Scale bar: 100 μm.

We recently reported on a decreased neurosphere formation capacity from primary cortical cells upon chlorate treatment [8]. Thus, we started to analyze the influence of NaClO_3_ on NPC proliferation by assaying the clonal neurosphere formation from E13.5 primary spinal cord cells. In order to investigate whether NaClO_3_ might just affect NPC subpopulations, we grew neurospheres under different growth factor conditions. Figure 3A,B shows representative pictures of neurospheres grown in the presence of EGF and FGF2 under control or NaClO_3_ conditions. Typical neurospheres of 100 μm to 200 μm diameter formed within one week under control conditions. In contrast, NaClO_3_-treated neurospheres were smaller. Moreover, the number of primary neurospheres was significantly reduced upon NaClO_3_ treatment (without: 75.6 ± 35.2; NaClO_3_: 33.8 ± 15.1; n = 5; *P* = 0.04; Figure 3C). In parallel, neurospheres were grown in high density cultures in the presence of EGF and FGF2, and the total cell number, as a measurement for the overall proliferation rate, was determined after one week. In the presence of NaClO_3_ we observed strongly reduced total cell numbers in the culture flasks (without: 2,240,000 ± 149,000; NaClO_3_: 622,000 ± 219,000; n = 4; *P* = 0.001; Figure 3D).

Next, we analyzed the influence of NaClO_3_ on EGF- or FGF2-dependent neurosphere formation. Again, the quantification revealed a general decrease in neurosphere formation capacity independently of the growth factor added (EGF only: 27.0 ± 6.9; EGF and NaClO_3_: 13.3 ± 3.5; n = 3; *P* = 0.034; FGF2 only: 63.0 ± 20.7; FGF2 and NaClO_3_: 17.0 ± 4.4; n = 3; *P* = 0.044; Figure 3E).

Finally, we further analyzed the effect of NaClO_3_ on the size of neurospheres grown in the presence of EGF and FGF2 for one week. For that purpose, photomicrographs of single neurospheres were used to measure the diameter of respective colonies. Data from four independent experiments revealed that the addition of NaClO_3_ resulted in a shift towards smaller neurospheres at the expense of larger neurospheres (Figure 3F). However, since there were still some large neurospheres in the presence of NaClO_3_, NaClO_3_ itself might only affect the proliferation rate of a subpopulation of NPCs.

### Sodium chlorate leads to G2-phase retention of spinal cord neural precursor cells

**Figure 4:**
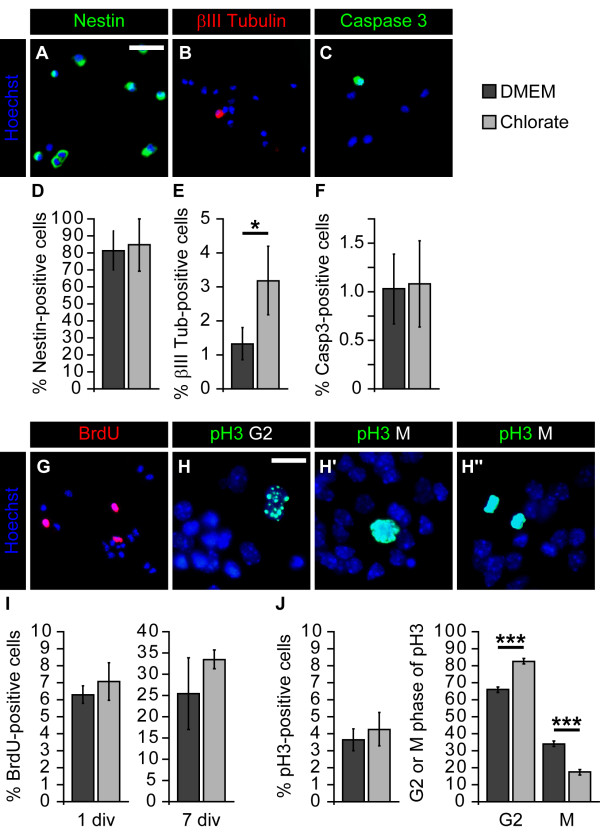
**Sodium chlorate induces G2 arrest of spinal cord neural precursor cells. (A-F)** Neurospheres grown as high density culture in the presence of EGF and FGF2 for one week were dissociated, and single cells were plated for 2 h on a poly-DL-ornithine substrate for further analysis. The percentage of Nestin-positive cells was not altered upon NaClO_3_ addition (n = 6). In contrast, the relative number of βIII-Tubulin-positive neurons was significantly enhanced (n = 5; *P* <0.05). NaClO_3_ did not affect the proportion of Caspase3-positive cells (n = 4). **(G, I)** BrdU incorporation analysis was performed after one day and after seven days. NaClO_3_ did not affect the BrdU incorporation rate for either time point investigated (1div: n = 4; 7div: n = 5). **(H, J)** Moreover, the relative number of pH3-positive cells was also not changed in response to NaClO_3_. Strikingly, the percentage of cells in the G2 and M-phase of the cell cycle was increased and decreased, respectively (n = 4; *P* = 0.0002). Hoechst 33528 was added to visualize the cell nuclei. Scale bar: 25 μm (H-H”), 50 μm (A, B, C, G).

In order to further analyze the influence of NaClO_3_ on NPC proliferation, we examined the cellular composition of neurospheres grown for one week in the presence of EGF and FGF2. The percentage of Nestin-positive cells was not altered (without: 82.2 ± 11.7%; NaClO_3_: 85.6 ± 15.5%; n = 6; Figure 4A,D). In contrast, the relative number of βIII-Tubulin-positive neurons was significantly increased upon NaClO_3_ treatment (without: 1.3 ± 0.5%; NaClO_3_: 3.2 ± 1.0%; n = 5; *P* = 0.014; Figure 4B,E). Finally, we did not observe changes in the percentage of Caspase3-positive cells undergoing apoptotic cell death (without: 1.03 ± 0.36%; NaClO_3_: 1.08 ± 0.44%; n = 4; Figure 4C,F).

Next, we added 1 μM BrdU for 1 h after one day and after one week to investigate the overall proliferation rate. Interestingly, the relative number of BrdU-positive cells was not changed for either time point investigated (without 1div: 6.3 ± 0.5%; NaClO_3_ 1div: 7.1 ± 1.1% (n = 3); without 7div: 25.4 ± 8.4%; NaClO_3_ 7div: 33.5 ± 2.2%; (n = 5); Figure 4G,I), indicating that the S-phase entry was not affected by NaClO_3_. We next analyzed both the percentage of pH3-positive cells after one day and their pH3 staining pattern, to assay for the G2- and M-phase (Figure 4H-H”) [28]. The relative amount of pH3-positive cells was not affected by NaClO_3_ (without: 3.6 ± 0.6%; NaClO_3_: 4.3 ± 1.0%; n = 3; Figure 4J). However, significantly more cells remained in the G2-phase after NaClO_3_ addition, while only a minority of pH3-positive cells proceeded into the M-phase (without G2: 66.0 ± 1.6%; NaClO_3_ G2: 82.6 ± 1.7%; (n = 4; *P* = 0.0002); without M: 34.0 ± 1.6%; NaClO_3_ M: 17.4 ± 1.7%; (n = 4; *P* = 0.0002); Figure 4J). These data indicate that NPCs grown in the presence of NaClO_3_ display a G2-phase arrest.

### Sodium chlorate affects Erk-signaling

**Figure 5:**
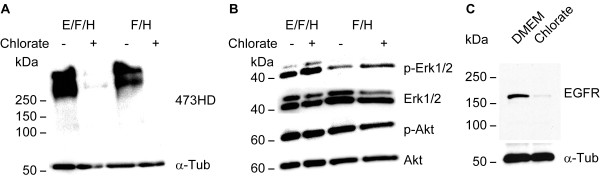
**Sodium chlorate increases Erk1/2 activation levels in primary neurospheres. (A)** Treatment of primary neurospheres grown either in the presence of EGF and FGF2 or FGF2 alone with NaClO_3_ reduced the expression level of the sulfation-dependent 473HD epitope. **(B)** In contrast, the phosphorylation of Erk1/2 (pErk1/2) was enhanced in response to NaClO_3_. However, NaClO_3_ did not affect the phosphorylation of Akt (pAkt), another downstream effector molecule of tyrosine kinase receptors. **(C)** In line with an enhanced spontaneous neuronal differentiation, the expression level of the EGFR was also reduced.

To gain first insights into potential molecular mechanisms mediating the cell biological influence of NaClO_3_ on spinal cord NPCs, we analyzed the activation levels of Erk1/2 and Akt, two effector molecules downstream of tyrosine kinase receptors. For that purpose, we used neurospheres from high density cultures grown in the presence of either EGF and FGF2 or FGF2 alone. Both conditions were additionally supplemented with heparin. The addition of NaClO_3_ resulted in a reduced expression of the 473HD epitope (Figure 5A), confirming the functionality of NaClO_3_ in that experiment. While the level of phosphorylated Akt (pAkt) was not changed, we observed increased levels of phosphorylated Erk1/2 (Figure 5B). Moreover, the overall expression level of the EGFR in the neurospheres was reduced upon NaClO_3_ treatment (Figure 5C). This was in line with the increased percentage of neurons in the presence of NaClO_3_.

### Sodium chlorate affects neuron numbers under differentiating conditions

Since CS-GAG chains regulate the differentiation of cortical NPCs [4-6], we next analyzed the impact of NaClO_3_ on spinal cord NPC differentiation upon growth factor withdrawal. For this purpose, neurospheres grown as high density cultures were dissociated and single cells were initially plated on a poly-DL-ornithine/laminin substrate for four days in the presence of 1 % FCS. The differentiation was immunocytochemically investigated using the cell type specific markers Nestin, GFAP, O4 and βIII-Tubulin. The general differentiation level was low after four days, since the majority of cells were still Nestin-positive (Additional file 2: Figure S2A). However, several GFAP-positive astrocytes as well as βIII-Tubulin-positive neurons and O4-positive immature oligodendrocytes were also present under both conditions (Additional file 2: Figure S2A, B). Again, counting for the different cell types revealed that the treatment with NaClO_3_ resulted in a higher percentage of βIII-Tubulin-positive neurons (without: 5.5 ± 1.6%; NaClO_3_: 8.8 ± 3.4%; n = 7; *P* = 0.041) (Additional file 2: Figure S2D). In contrast, neither astroglial nor oligodendroglial differentiation were significantly affected after four days of differentiation (GFAP without: 3.3 ± 2.6%; GFAP with NaClO_3_: 5.5 ± 4.2%; n =7; O4 without: 1.9 ± 0.6%; O4 with NaClO_3_: 1.6 ± 1.2%; n = 7) (Additional file 2: Figure S2C, E).

### Sodium chlorate affects morphological and functional maturation of neural precursor cell-derived neurons

**Figure 6:**
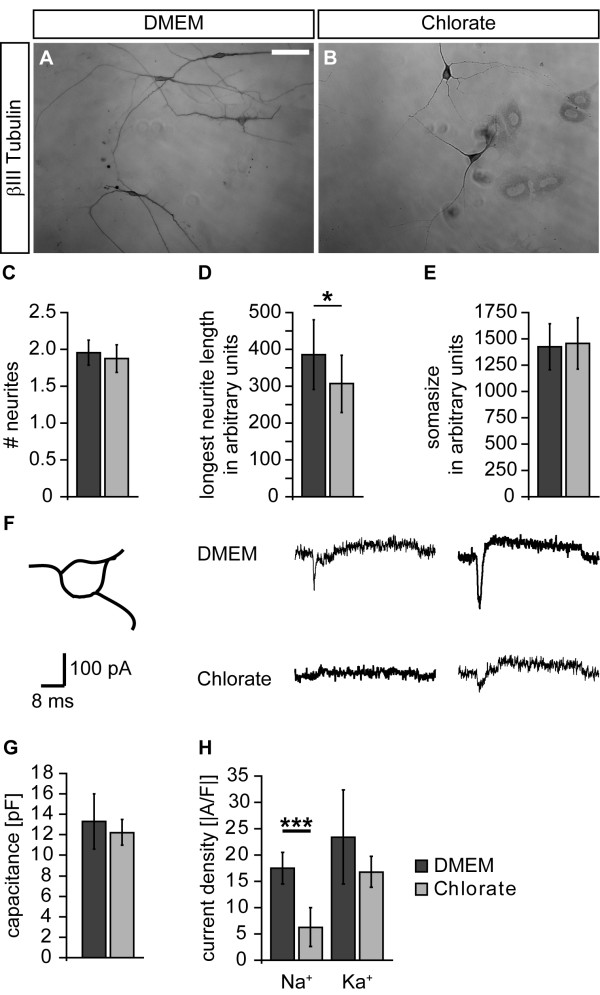
**Sodium chlorate affects morphological and functional maturation of neural precursor cell-derived neurons. (A, B)** In order to analyze the neuronal morphology, the βIII-Tubulin immunoreactivity was visualized using diaminobenzidine. In the control situation the neurons often displayed a multipolar morphology with long neurites. Upon NaClO_3_ treatment the neurites appeared to be shorter. **(C-E)** To assess the morphological maturation, the neurite number, the length of the longest neurite and the soma size were measured. The latter two are given in arbitrary units. While the neurite number and the soma size were not altered in the presence of NaClO_3_ (n = 6), the mean length of the longest neurite was significantly reduced (n = 6; *P* <0.05). **(F)** Whole cell patch-clamp recordings of individual NPC-derived neurons were performed under voltage clamp conditions. To take neuronal subpopulations into account, only neurons exhibiting a triangular soma shape were analyzed. Upon depolarization, the neurons responded with a brief sodium inward current followed by a delayed potassium outward current. The inward component was strongly reduced in the presence of NaClO_3_. **(G)** The determination of the cell capacitance revealed no changes in the overall cell size upon NaClO_3_ treatment (n = 5 preparations). **(H)** Analysis of the two current components expressed as current density clearly showed a reduced sodium current density in the presence of NaClO_3_ (n = 5 preparations; *P* <0.001). In contrast, the potassium current density was not affected (n = 5 preparations). Scale bar: 25 μm.

The observation that neuronal differentiation was enhanced in the presence of the sulfation inhibitor NaClO_3_ prompted us to further examine neuronal maturation with regard to morphology. To address that issue, we plated dissociated primary neurosphere cells under differentiating conditions and analyzed the neuronal morphology after four days in terms of neurite number, length of the longest neurite and basal soma size. Figure 6A,B shows representative photomicrographs of neurons grown in the absence and presence of NaClO_3_. The mean number of neurites was not affected by NaClO_3_ (without: 1.95 ± 0.17; NaClO_3_: 1.88 ± 0.19; n = 6; Figure 6C). In contrast, the mean length of the longest neurite was significantly reduced upon NaClO_3_ treatment (without: 385.5 ± 94.6; NaClO_3_: 306.8 ± 77.8; n = 6; *P* = 0.044; Figure 6D). The average soma size was also not changed (without: 1,426 ± 218; NaClO_3_: 1,454 ± 253; n = 6; Figure 6E).

Next, we analyzed if NaClO_3_ also affects electrophysiological properties of NPC-derived neurons. Thus, we performed whole cell patch-clamp recordings of NPC-derived neurons. To take potential neuronal subpopulations into consideration, we specifically analyzed neurons with a triangular cell soma (Figure 6F). Upon supra-threshold depolarization, the neurons usually responded with an inward current (sodium current component) followed by a prolonged outward current (potassium current component). Following culture in NaClO_3_, peak sodium currents were strongly decreased in comparison to cells cultured under control conditions (Figure 6F). To ensure that these differences were not due to differences in cell size, we first determined the cell capacitance and did not observe any changes in response to NaClO_3_ treatment (without: 13.3 ± 2.7 pF; NaClO_3_ ± 12.2 ± 1.3 pF; n = 5 preparations; Figure 6G). Then, we measured both the sodium inward and potassium outward current and normalized both components to the cell capacitance, yielding current densities. We found that the sodium current density was significantly reduced in the presence of NaClO_3_ (without: -17.5 ± 2.9 A/F; NaClO_3_: -6.3 ± 3.6 A/F; n = 5 preparations; *P* <0.001; Figure 6H). In contrast, the potassium current density was not significantly changed (without: 23.4 ± 8.9 A/F; NaClO_3_: 16.8 ± 3.0 A/F; n = 5 preparations; Figure 6H).

**Figure 7:**
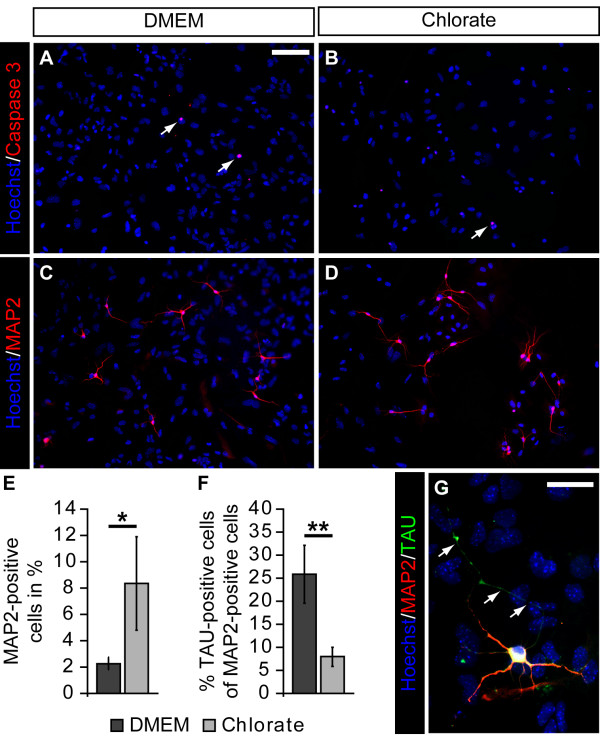
**Sodium chlorate attenuates neuronal polarization of neural precursor cell-derived neurons. (A-D, G)** Photomicrographs of differentiated NPCs stained for cleaved Caspase3, MAP2, and Tau after seven days of differentiation. **(A-D)** NaClO_3_ treatment did not result in an increased apoptotic cell death after seven days. However, in line with an increased percentage of βIII-Tubulin-positive neurons after four days, the relative number of MAP2-positive cells was enhanced in response to NaClO_3_. **(E)** The quantification of MAP2-positive neurons revealed a significant increase in the relative amount of neurons in NaClO_3_ treated cultures (n = 4; *P* = 0.015). **(F, G)** Interestingly, the percentage of Tau-positive cells among MAP2-positive neurons was strongly reduced after NaClO_3_ treatment (n = 4; *P* = 0.002). To visualize the nuclei the cells were counterstained with Hoechst 33528. Scale bar: 50 μm (A-D), 25 μm (G).

To further analyze the influence of NaClO_3_ on neuronal maturation, we extended the differentiation period for another three days. After that, we first determined the relative number of Caspase3-positive cells to investigate whether potential differences might be due to changes in the rate of apoptotic cell death. However, we did not notice any differences in the percentage of Caspase3-positive cells (Figure 7A,B). Next, we counted the number of MAP2-positive cells and found a significant increase in the percentage of MAP2-positive cells upon NaClO_3_ treatment (without: 2.3 ± 0.5%; NaClO_3_: 8.4 ± 3.6% (n = 4; *P* = 0.015)). Finally, we analyzed the polarization of the neurons in our differentiation assay by staining for MAP2 (dendritic compartment) in combination with Tau (axonal compartment). While under control conditions, a substantial number of MAP2-positive cells also had a Tau-positive axon; the relative number of clearly polarized neurons in the presence of NaClO_3_ was significantly reduced (without: 25.9 ± 6.3%; NaClO_3_ 8.0 ± 2.1%; (n = 4; *P* = 0.002)). We also observed a similar phenomenon in two independent experiments after a ten-day differentiation period (data not shown).

## Discussion

GAG chains on proteins of the extracellular matrix or on membrane bound cell adhesion molecules are complex carbohydrates that participate in many biological processes during development and in adulthood, in part via their highly specific sulfation patterns. Sulfation of GAGs is performed by sulfotransferases that have been shown to be particularly relevant in developmental processes [8,29-34]. In this study, we initially investigated the expression of the 473HD-epitope, a unique CS-motif, during embryonic spinal cord development and found that it is expressed at late embryonic ages. Its pronounced expression within the NPC compartment, moreover, suggests a functional role of sulfated GAG chains in the regulation of spinal cord NPC biology. Along these lines it has been shown that highly sulfated complex GAG structures are expressed by cortical NPCs [35-37] and regulate proliferation and differentiation of embryonic and adult cortical NPCs [4-7,38]. Thus, we analyzed the role of changes in sulfation on the proliferation and differentiation potential of mouse embryonic spinal cord NPC using NaClO_3_ as a potent inhibitor of eukaryotic sulfation reactions. Low concentrations of NaClO_3_ (2 mM to 5 mM) preferentially inhibit sulfation of CS-GAGs, whereas higher concentrations (15 mM to 30 mM) are required to inhibit sulfation of HS-GAGs as well [39]. We used a concentration of 30 mM, to assure an almost complete sulfation blockage of both CS-GAGs and HS-GAGs. To monitor the effectiveness of NaClO_3_, we analyzed the expression level of the 473HD-epitope, and observed an almost complete reduction of the epitope after NaClO_3_ treatment. This observation is in line with a former study demonstrating that the 473HD-epitope depends on a distinct sulfation pattern [40]. Some residual 473HD immunoreactivity in the western blot as well as in the immunohistochemical analyses is likely due to the fact that NaClO_3_ might not affect every neurosphere cell as soon as the neurospheres reach a certain size. However, since the total amount of the carrier protein RPTPβ/ζ does not change, our data prove the functionality of NaClO_3_ to specifically affect sulfation events.

We next analyzed clonal neurosphere formation capacity from E13.5 spinal cord NPCs. Treatment of neurosphere cultures with 30 mM NaClO_3_ resulted in significantly fewer and smaller neurospheres. This effect was independent from the growth factor used to propagate the neurospheres (EGF, FGF2 or both; Figure 4 C-E). Looking at the diameter of the spheres, it became obvious that there are more small-diameter neurospheres and fewer large-diameter neurospheres when the cultures were treated with NaClO_3_ (Figure 4A,B,F). This phenomenon has been observed previously for the embryonic telencephalic neurospheres [8]. The reasons for the altered neurosphere growth could be that NPCs within the neurosphere divide more slowly in the presence of NaClO_3;_ NPCs die more frequently; or NPCs have a higher differentiation capacity, and therefore NaClO_3_ might affect stem or precursor cell maintenance. Our BrdU incorporation analysis did not reveal any changes in the S-phase of the cell cycle. However, while the relative number of pH3-positive cells present in the G2-phase of the cell cycle was significantly enhanced, the percentage of M-phase cells was significantly decreased, indicating a potential G2-phase arrest in the presence of NaClO_3_. Although a G2-arrest is a common phenomenon preceding apoptotic cell death, especially in cancer cells [41], we did not observe an increased percentage of Caspase3-positive cells, Thus, it is unlikely that NaClO_3_ affected cell death rates in our culture system. Besides the difference with regard to the G2/M-phase of the cell cycle, we also documented a significant increase of βIII-Tubulin-positive young neurons in response to NaClO_3_ treatment. Furthermore, the overall expression level of EGFR in the neurosphere cultures was reduced. This is also in line with an increased percentage of βIII-Tubulin-positive neurons, since the acquisition of the EGFR is generally considered a hallmark for the developmental switch from neurogenic NPCs towards gliogenic NPCs [42]. However, based on our data, we conclude that NaClO_3_ primarily affects spinal cord NPC proliferation by changing the cell cycle kinetics. This results in a higher number of neurons already under proliferative conditions. However, it remains unsolved whether NaClO_3_ truly promotes neurogenic differentiation. It might be that the enhanced percentage of neurons simply reflects the reduced proliferation capabilities of the surrounding cells.

Regarding potential molecular mechanisms, we initially reasoned that the suppression of sulfation might compromise growth factor signaling, leading to changes in the activation levels of downstream effector molecules such as Erk1/2 and Akt. Surprisingly, we observed an increased Erk1/2 activation in the presence of NaClO_3_. Yet an increased Erk1/2 activation has already been reported previously in the context of a G2 arrest in different cancer cell lines [43,44]. It is unlikely that the increased Erk1/2 activation depends on increased receptor activation, because the lack of sulfation should result in a reduced growth factor signaling strength. Along these lines, we recently showed that the enzymatic degradation of CS-GAGs compromises FGF signaling in cortical neurosphere cultures, resulting in a reduced MAP kinase activation [5]. Here, we think that NaClO_3_ might reduce the activation levels of phosphatases through a yet unknown mechanism. In this context, it is noteworthy that RPTPβ/ζ bears sulfated CS-GAGs [25] and regulates Erk1/2 activation levels in human keratinocytes [45]. Therefore, the sulfation status of RPTPβ/ζ might have a critical influence on its activation level and as a consequence also on the Erk1/2 activation levels.

Our laboratory has recently shown that degradation of CS-GAGs from telencephalic NPCs using ChABC results in a pronounced glial differentiation at the expense of neuronal cell types [6]. With regard to spinal cord NPCs, we observed a higher proportion of neuronal cells already under proliferative conditions. Under differentiating conditions we documented an enhanced relative number of βIII-Tubulin-positive cells after four days and MAP2-positive cells after one week following NaClO_3_ treatment. These data are in line with previous studies demonstrating an increased neuronal differentiation of mouse embryonic stem cells upon siRNA mediated knock down of either the PAPS transporters 1 and 2 or HS-specific sulfotransferases [46]. In contrast to the increased percentage of neurons, the relative number of glial cells was not affected.

To further elucidate the phenotype of the βIII-Tubulin-positive cells, we took a closer look at the morphology of these cells and valued the morphology as a level of maturation. The spinal cord NPC-derived neurons exhibited no changes with respect to soma size and total primary neurite number upon NaClO_3_ treatment. But they significantly differed in the length of their longest neurite. This was surprising, since CSPGs and HSPGs are primarily known for their inhibitory influence on neurite outgrowth, particularly under pathological conditions [10,47]. Moreover, CS-GAGs have recently been shown to influence the morphology of NPC-derived neurons in terms of neurite number, length and branching [4,48]. Yet there is growing evidence that specific CS-GAGs also promote neurite outgrowth depending on both the mode of their presentation (that is, homogenous substrate or alternating substrate) and on the neuronal cell type [13,25,49]. Thus, we believe that our data hint towards an attenuated maturation of spinal cord NPC-derived neurons in the presence of NaClO_3_*in vitro*. In line with this hypothesis, we also documented a strongly reduced neuronal polarity after seven and ten days in culture. To check whether this delayed morphological maturation is also relevant at a functional level, we analyzed the differentiated neurons grown from NaClO_3_-treated dissociated cultures in comparison with the control group using whole cell patch-clamp recordings. We determined that the overall cell capacitance of the cells was not changed. This is in line with the fact that the soma size was not changed either. However, we observed a significantly reduced sodium current density, while the potassium current density remained unaltered. Lower sodium currents suggest a delay in functional neuronal maturation, because sodium currents usually increase upon differentiation of neuronal precursors during embryogenesis [50]. Furthermore, neurite outgrowth and sodium channel- dependent excitability have previously been shown to be tightly coupled during functional neuronal maturation [51]. Interestingly, the addition of FGF2 to rat postnatal hippocampal neurons significantly enhances the sodium current density, demonstrating a growth factor signaling-dependent regulation [52]. This might be mediated by highly sulfated GAG chains. In this context, it is noteworthy that we recently demonstrated changes in spontaneous synaptic activity of primary hippocampal neurons upon ChABC-mediated CS-GAG degradation [53].

## Conclusions

Based on our data, we believe that normal sulfation levels of CSPGs and HSPGs during spinal cord histogenesis promote cellular maturation in general and neuronal maturation in particular. Since both CSPGs and HSPGs also show a pronounced expression under pathological conditions, our data particularly highlight the need for a better understanding of the impact of specific PGs and their sulfation patterns for neural development and regeneration.

## Abbreviations

AKT, Protein kinase B (also PKB); BLBP, Brain lipid binding protein; BrdU, 5-bromo2’-deoxy-uridine; CNS, Central nervous system; CSPG, Chondroitin sulfate proteoglycan; Div, Day(s) in vitro; E0.5, E9.5, E12.5, E18.5, Embryonic day 0.5, 9.5, 12.5, 18.5; EGF, Epidermal growth factor; EGFR, Epidermal growth factor receptor; ERK, Extracellular signal-regulated protein kinase; FGF, Fibroblast growth factor; FGFR, Fibroblast growth factor receptor; GAG, Glycosaminoglycan; GFAP, Glial fibrillary acidic protein; GLAST, Glutamate Aspartate transporter; H, Hour(s); HRP, Horse-reddish peroxidase; MAP2, Microtubule-associated protein 2; mM, Milli Molar; NaClO3, Sodium Chlorate; NPC, Neural precursor cell; PBS, Phosphate-buffered saline; PG, Proteoglycan; pH3, Phosphor-Histone 3; RPTP, Receptor protein tyrosine phosphatase.

## Competing interest

The authors declare that they have no competing interests.

## Authors’ contributions

MK carried out the stem cell cultures and characterization and drafted the manuscript. SaSa participated in the neural stem cell characterization and performed statistical analyses. CB carried out the electrophysiological experiments. TT did the western blot analysis of the signaling pathways. ID designed the electrophysiological experiments. AF designed part of the immunohistochemical experiments. SW conceived the study and helped drafting the manuscript. All authors read and approved the final manuscript.

## Supplementary Material

Additional file 1 **Figure S1.** CS-GAGs are expressed during early gliogenesis of mouse spinal cord development. (A-E) Photomicrographs of frontal spinal cord sections stained against the 473HD epitope. At the beginning of neurogenesis (E9.5) the 473HD epitope was mainly expressed in the meninges and the surrounding parenchyma. Towards the end of neurogenesis (E12.5) the 473HD epitope appeared at the ventral central canal. One day later the immunoreactivity has expanded into the prospective ventral white matter and to a less extent into the dorsal spinal cord. Note that both the floor plate and the roof plate lacked any immunoreactivity. At E15.5 the immunoreactivity was even stronger in the ventral spinal cord and still low in the dorsal part. Another three days later the 473HD epitope was evenly distributed throughout the whole spinal cord except for the region around the central canal. To visualize the nuclei, the cells were counterstained with Hoechst 33528. Scale bar: 50 μm (A, B), 100 μm (C-D). Click here for file

Additional file 2 **Figure S2.** Sodium chlorate promotes neuronal differentiation of spinal cord NPCs. (A, B) After one week in the presence of EGF and FGF2 neurospheres were dissociated, and the differentiation capacity was immunocytochemically analyzed using cell type specific markers. The overall differentiation was low, since about 75% of all cells still expressed the NPC marker Nestin after that time period. (C) The quantification of GFAP-positive astrocytes showed no differences between the NaClO_3_ and control conditions (n = 7). (D) However, the number of βIII-Tubulin-positive neurons was significantly increased in the presence of NaClO_3_ (n = 7; *P* <0.05). (E) The amount of O4-positive immature oligodendrocytes was not changed (n = 7). Hoechst 33528 was added, to visualize the cell nuclei. Scale bar: 50 μm. Click here for file
