# Supplementary figures and images for: Normal sulfation levels regulate spinal cord neural precursor cell proliferation and differentiation
Source: Neural Dev. 2012 Jun 8;7:20. doi: 10.1186/1749-8104-7-20 (PMC3423038; doi:10.1186/1749-8104-7-20)

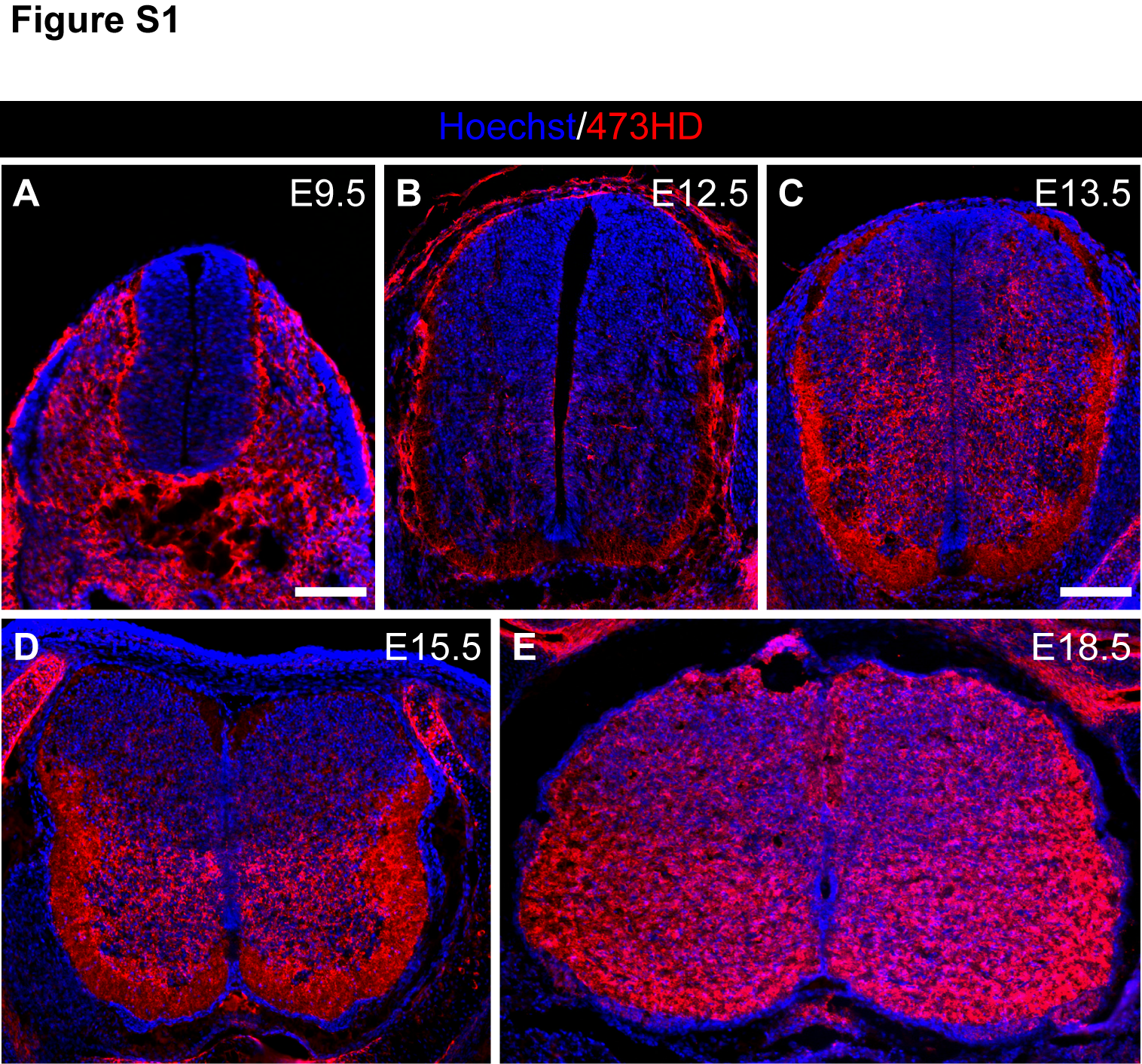

Supplement: Additional file 1 — Figure S1. CS-GAGs are expressed during early gliogenesis of mouse spinal cord development. (A-E) Photomicrographs of frontal spinal cord sections stained against the 473HD epitope. At the beginning of neurogenesis (E9.5) the 473HD epitope was mainly expressed in the meninges and the surrounding parenchyma. Towards the end of neurogenesis (E12.5) the 473HD epitope appeared at the ventral central canal. One day later the immunoreactivity has expanded into the prospective ventral white matter and to a less extent into the dorsal spinal cord. Note that both the floor plate and the roof plate lacked any immunoreactivity. At E15.5 the immunoreactivity was even stronger in the ventral spinal cord and still low in the dorsal part. Another three days later the 473HD epitope was evenly distributed throughout the whole spinal cord except for the region around the central canal. To visualize the nuclei, the cells were counterstained with Hoechst 33528. Scale bar: 50 μm (A, B), 100 μm (C-D). [file 1749-8104-7-20-S1.tiff]

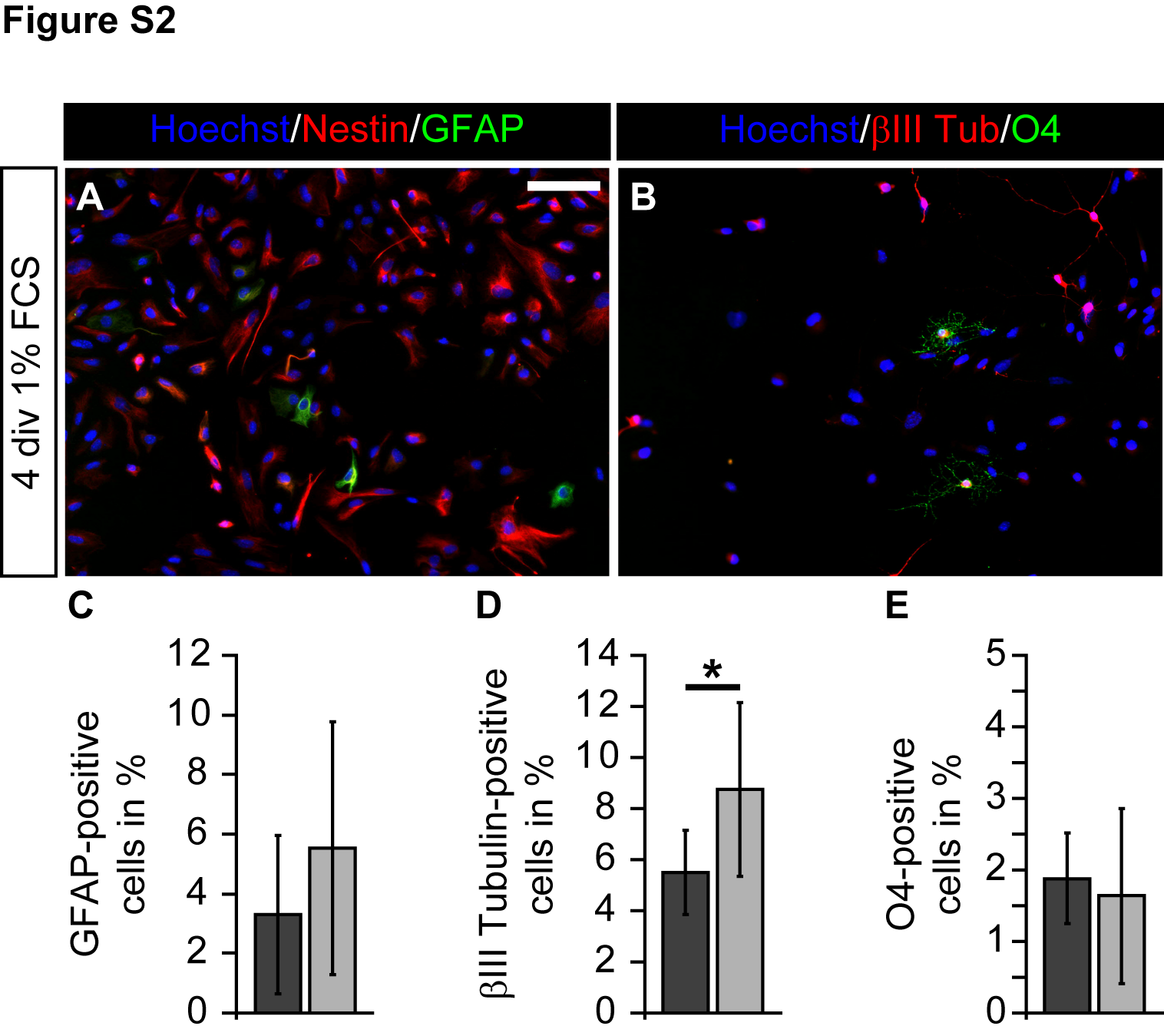

Supplement: Additional file 2 — Figure S2. Sodium chlorate promotes neuronal differentiation of spinal cord NPCs. (A, B) After one week in the presence of EGF and FGF2 neurospheres were dissociated, and the differentiation capacity was immunocytochemically analyzed using cell type specific markers. The overall differentiation was low, since about 75% of all cells still expressed the NPC marker Nestin after that time period. (C) The quantification of GFAP-positive astrocytes showed no differences between the NaClO3 and control conditions (n = 7). (D) However, the number of βIII-Tubulin-positive neurons was significantly increased in the presence of NaClO3 (n = 7; P <0.05). (E) The amount of O4-positive immature oligodendrocytes was not changed (n = 7). Hoechst 33528 was added, to visualize the cell nuclei. Scale bar: 50 μm. [file 1749-8104-7-20-S2.tiff]
